# Relationship between promyelocytic leukemia protein nuclear bodies and TAR DNA-binding protein-43 aggregation in spinal anterior horn cells in sporadic amyotrophic lateral sclerosis

**DOI:** 10.1093/jnen/nlag029

**Published:** 2026-04-02

**Authors:** Fumiaki Mori, Tomoya Kon, Riho Itazawa, Ami Akatsu, Yasuo Miki, Akira Arai, Hidekachi Kurotaki, Masahiko Tomiyama, Koichi Wakabayashi

**Affiliations:** Department of Neuropathology, Biomedical Research Center, Hirosaki University Graduate School of Medicine, Hirosaki, Japan; Department of Neurology, Hirosaki University Graduate School of Medicine, Hirosaki, Japan; Department of Neurology, Hirosaki University Graduate School of Medicine, Hirosaki, Japan; Department of Neuropathology, Biomedical Research Center, Hirosaki University Graduate School of Medicine, Hirosaki, Japan; Department of Neuropathology, Biomedical Research Center, Hirosaki University Graduate School of Medicine, Hirosaki, Japan; Department of Neurology, Aomori Prefectural Central Hospital, Aomori, Japan; Department of Pathology, Aomori Prefectural Central Hospital, Aomori, Japan; Department of Neurology, Hirosaki University Graduate School of Medicine, Hirosaki, Japan; Department of Neuropathology, Biomedical Research Center, Hirosaki University Graduate School of Medicine, Hirosaki, Japan

**Keywords:** anterior horn cell, nuclear body, PML, sporadic amyotrophic lateral sclerosis, TDP-43 inclusion

## Abstract

Promyelocytic leukemia protein nuclear bodies (PML-NBs) and stress granules serve as deposition sites for stress-induced, aggregation-prone proteins. We previously reported that TAR DNA-binding protein 43 (TDP-43) colocalizes with stress granules during early aggregation in sporadic amyotrophic lateral sclerosis (ALS), and recent studies have noted PML-NB loss in familial ALS. To explore the role of PML-NBs in TDP-43 inclusion maturation, we analyzed spinal cord specimens from 12 patients with sporadic ALS and 5 controls using immunostaining for PML and TDP-43. PML-NB counts in anterior horn cells (AHCs) were significantly lower in patients with ALS than in controls (*P* < 0.05), especially in AHCs with TDP-43 inclusions (*P* < 0.01). Average numbers of PML-NB decreased progressively with inclusion type (3.1 in diffuse punctate cytoplasmic staining, 2.3 in round inclusions, and 0.8 in skein-like inclusions); all of these were significantly lower than those in inclusion-free AHCs (controls: 4.6; ALS: 5.5; *P *< 0.01). AHCs in ALS without inclusions showed higher PML-NB counts than in controls (*P *< 0.05), suggesting an early protective response. In contrast, reduced PML-NBs in mature inclusions may reflect diminished cellular defense. These findings implicate PML-NBs in the pathogenesis of sporadic ALS.

## INTRODUCTION

Amyotrophic lateral sclerosis (ALS) is a neurodegenerative disease caused by interactions between genetic and external environmental factors. To date, more than 20 genes have been linked to familial ALS.^1^ Sporadic ALS occurs spontaneously in individuals with no apparent family history and accounts for approximately 90% of all ALS cases. Pathologically, ALS is characterized by the loss of upper and lower motor neurons with the frequent occurrence (>95%) of TAR DNA-binding protein 43 (TDP-43) inclusions. TDP-43 is an RNA-binding protein that is predominantly localized in the nucleus under normal conditions and has multiple functions in RNA metabolism. This protein is also present in the cytoplasm and shuttles between the nucleoplasm and cytoplasm. In patients with ALS, TDP-43 undergoes abnormal phosphorylation (p-TDP-43) in the cytoplasm, leading to conformational changes that eventually lead to the formation of TDP-43 inclusions. A growing body of evidence suggests that nuclear-to-cytoplasmic mislocalization of physiological TDP-43 or abnormal aggregation of p-TDP-43 induces toxicity via both loss- and gain-of-function mechanisms. Recently, Kon et al reported that diffuse punctate cytoplasmic staining (DPCS) is the earliest manifestation of TDP-43 pathology in sporadic ALS and that DPCS corresponds to nonfibrillar TDP-43 located at the ribosomes of the rough endoplasmic reticulum.^2^ Moreover, we previously reported that TDP-43 colocalizes with stress granules (SGs) in the early phase of TDP-43 aggregation in sporadic ALS.^3^

Previous studies using cultured cells have suggested that TDP-43 inclusions are derived from degenerated SGs, based on the presence of numerous SG markers in the inclusions.^4^ SGs are dynamic, reversible biomolecular condensates that accumulate in the cytoplasm of eukaryotic cells under various stressful conditions, including oxidative, osmotic, and heat stress, ultraviolet radiation, and viral infections.^5–7^ They range from 0.2 to 1 μm in size and are composed mainly of SG-marker proteins and non-translating mRNAs.^8^ SG formation typically occurs upon stress-induced translational arrest and polysome disassembly.^9^

Cytoplasmic SGs and promyelocytic leukemia protein nuclear bodies (PML-NBs) are deposition sites for stress-induced aggregation-prone proteins. PML-NBs are multiprotein structures organized in a core–shell manner^10^ with a size ranging from 0.2 to 1 μm, distributed in the nucleus (up to 30 bodies per nucleus).^11^^,^^12^ Oxidative stress inducers such as arsenic trioxide regulate PML-NB formation. Other in vivo oxidative stress-inducing agents, such as doxorubicin, paraquat, and irradiation, induce an increase in the number of PML-NBs.^13^ Although apparently unrelated, cytoplasmic SGs and PML-NBs are interconnected via the small ubiquitin-related modifier (SUMO)-targeted ubiquitin ligase pathway, in which 2 post-translational modifications, SUMOylation and ubiquitylation, and RNF4 E3 ligase collaborate to clear damaged proteins.^14^ The loss of PML-NBs has been recently reported in patients with familial ALS.^15^ However, the role of PML-NBs in the pathogenesis of sporadic ALS remains unclear. Therefore, we investigated the association between PML-NBs and TDP-43 aggregation in the anterior horn cells (AHCs) of patients with sporadic ALS.

## METHODS

### Participants

This study included 12 patients with sporadic ALS and 5 healthy controls (Table 1). All patients with ALS had been diagnosed, followed up, and died at the Hirosaki University Hospital or Aomori Prefectural Central Hospital. The pathological diagnosis of ALS was confirmed by the loss of upper and lower motor neurons and the presence of p-TDP-43-immunoreactive neuronal cytoplasmic inclusions (NCIs). Considering the natural history of ALS,^16^ we designated ALS cases with a disease duration ≤ 1 year as short-duration ALS (n = 5) and those with a disease duration of 2 to 5 years as standard-duration ALS (n = 7). Five age- and sex-matched control participants were also enrolled (Table 1). The clinical and neuropathological findings in 2 patients with ALS (Patients 3^17^ and 4^18^) have been reported previously.

**Table 1.** Subjects.

No.	Age at death (years)	Sex	Diagnosis	Disease duration (months)	Nishihira type	No. of AHCs	No. of AHCs with Nuclei	％	Average No. of PML-NBs	No inclusion	DPCS	RIs	SLIs	Inclusions (DPCS+RIs+SLIs)
**Short duration**														
**1**	61	M	ALS-D	5	2	148	59	39.9	3.9	22	17	18	2	37
**2**	42	M	ALS	7	1	97	50	51.5	4.1	16	27	6	1	34
**3**	68	M	ALS+CIDP	11	1	44	24	54.5	2.9	13	8	1	2	11
**4**	76	F	ALS	12	2	101	45	44.6	5.7	16	18	10	1	29
**5**	77	M	ALS	12	1	65	26	40.0	3.5	11	4	10	1	15
**Average/Total**	64.8			8.8		455	204	46.1	4.0	78	74	45	7	126
**Standard duration**														
**6**	59	M	ALS-D	24	2	90	32	35.6	3.5	23	6	2	1	9
**7**	63	M	ALS	24	1	118	38	32.2	5.8	25	10	2	1	13
**8**	54	M	ALS	34	1	67	27	40.3	2.5	13	7	4	3	14
**9**	80	F	ALS	53	1	57	25	43.9	2.7	14	3	5	3	11
**10**	73	F	ALS	30	1	29	19	65.5	5.4	10	7	2	0	9
**11**	67	F	ALS	36	1	25	15	60.0	4.7	12	3	0	0	3
**12**	69	F	ALS	46	1	48	19	39.6	2.6	14	2	0	3	5
**Average/Total**	63.9			36.9		434	175	45.3	3.9	111	38	15	11	64
**Short+Standard**						889	379	42.6	4.1	189	112	60	18	190
**Control**														
**13**	64	M	Control	NA	NA	133	56	42.1	4.4	56	0	0	0	0
**14**	72	M	Control	NA	NA	152	63	41.4	4.5	63	0	0	0	0
**15**	40	M	Control	NA	NA	100	42	42.0	5.3	42	0	0	0	0
**16**	84	M	Control	NA	NA	105	43	41.0	4.1	43	0	0	0	0
**17**	83	F	Control	NA	NA	101	50	49.5	2.6	50	0	0	0	0
**Average/Total**	68.6					591	254	43.2	4.2	254	0	0	0	0

AHCs, anterior horn cells; PML-NBs, Promyelocytic leukemia protein nuclear bodies; DPCS, diffuse punctate cytoplasmic staining; RIs, round inclusions; SLIs, skein-like inclusions; ALS-D, ALS with dementia; CIDP, chronic inflammatory demyelinating polyneuropathy; NA, not applicable.

### Morphological analysis

We divided TDP-43-immunoreactive NCIs into 3 types, based on the study by Kon et al.^2^: (1) DPCS, viz. fine punctate granules scattered diffusely in the cytoplasm; (2) round inclusions (RIs), ie, large spherical inclusions approximately 1 to 15 µm in diameter; and (3) skein-like inclusion (SLIs), which are aggregates of thread-like structures. When DPCS was accompanied by SLIs or RIs, it was classified as an SLI or RI, respectively.

### Immunohistochemistry and morphometry

Three sections (4-µm thick) were cut serially from paraffin blocks of the fourth lumbar segment of the spinal cord in all cases and pretreated for heat-induced epitope retrieval for 10 minute in 10 mmol/L citrate buffer (pH = 6.0) using an autoclave. The first section was immunostained with rabbit anti-p-TDP-43 (TIP-PTD-P07; Cosmo Bio Co., Ltd, Tokyo, Japan; 1:5000), the second section with mouse anti-PML (PM001; MBL Co., Ltd, Nagoya, Japan; 1:500), and the third section with rabbit anti-native TDP-43 (n-TDP-43) (10782-1-AP; Proteintech, Chicago, IL; 1:2000), using the avidin-biotin-peroxidase complex method with diaminobenzidine as the chromogen. We analyzed 2 sets of 3 serial sections immunostained with anti-p-TDP-43, anti-PML and anti-n-TDP-43, respectively. To confirm PML immunoreactivity in PML-NBs, selected sections were also immunolabeled with another anti-PML antibody (ab179466, 1:100; Abcam), per the methodology of a recent study by Antoniani et al.^15^

Digital images of the anterior horn on both sides were captured using a digital camera (Provis AX-70; Olympus, Tokyo, Japan), enlarged, and printed on 3 sheets of A3 paper (×140). AHCs were defined as cells with a somatic diameter >37 µm in Rexed laminae VIII and IX.^19^ AHCs that contained nuclei were numbered on the enlarged prints. When the same neuronal cell body was recognized across 3 contiguous sections, the neuron was assigned identical numbers to ensure identification of the same AHC. Each AHC was scrutinized for PML-NBs and the latter were counted under a light microscope (BX-51; Olympus, Tokyo, Japan) equipped with an ocular lens (×10) and objective lens (×40). The numbers and densities of PML-NBs were evaluated in 4 groups of specimens: AHCs with DPCS, RIs or SLIs, or AHCs without inclusions (no inclusion). An interactive pen display with Image J/Fiji 1.46 software was used to measure the areas of the cell bodies, nuclei, and nucleoli of the AHCs on the digital images. The density of the PML-NBs was calculated by dividing the number of PML-NBs by the nuclear area. All samples were coded, and morphometric analyses were performed by 2 authors (FM and AA) who were blinded to the cases.

### Immunoelectron microscopy

Fifty-micrometer-thick vibratome sections of the lumbar cord obtained from 3 patients with ALS (Patients 1, 3, and 4) were incubated with rabbit polyclonal anti-p-TDP-43 antibody (TIP-PTD-P07, 1:500; Cosmo Bio Co., Ltd) for 2 days at 4°C, followed by incubation with a 1.4-nm gold-coupled Fab’ fragment of goat anti-rabbit IgG (1:50; Nanoprobes, Yaphank, NY). The sections were visualized using a silver-enhancing kit (BB International, Cardiff, UK), followed by treatment with osmium tetroxide (1% in 0.1 M phosphate buffer), block staining with uranyl acetate, dehydration in a series of graded ethanol solutions and flat-embedding on glass slides in Poly/Bed 812 Resin (Polysciences Inc., Warrington, PA). Regions containing target structures (DPCS, RIs or SLIs) were cut away, glued on a flat-surfaced plastic block, and cut into serial 0.5-μm-thick semithin sections. The sections were stained with toluidine blue to identify the immunolabeled target structures. After identifying the targets, the blocks were cut into 90-nm-thick ultrathin sections, which were stained with uranyl acetate and lead citrate and viewed under a JEOL1230 transmission electron microscope (JEOL Ltd, Tokyo, Japan).

### Statistical analysis

The one-way analysis of variance (ANOVA) with the Tukey–Kramer post hoc method was used to assess the differences in the numbers of PML-NBs and the areas of cell bodies, nuclei, and nucleoli in the AHCs between patients with ALS and normal controls. The 2-way ANOVA was used to assess differences in the number of PML-NBs between short- and standard-duration ALS. The Pearson correlation coefficient test was performed to assess the relationship between disease duration and the number of surviving AHCs. The Fisher exact probability test was used to assess the relationship between the formation of p-TDP-43-positive inclusions and the appearance of intranuclear vacuoles. Calculations were performed using Statcel software (OMS Publishing, Tokyo, Japan). Statistical significance was set at *P* < 0.05.

### Ethical approval

This study was conducted in accordance with the Declaration of Helsinki and was approved by the Institutional Ethics Committee of Hirosaki University Graduate School of Medicine, Hirosaki, Japan. Written informed consent was obtained from all participants’ families.

## RESULTS

### Clinical and histopathological findings

The clinical and histopathological findings are shown in Table 1. Since the average number of PML-NBs did not differ significantly between standard- and short-duration ALS (Figure S1), we analyzed these cases as a single group.

### TDP-43 and PML immunoreactivity of AHCs

**Figure 1:**
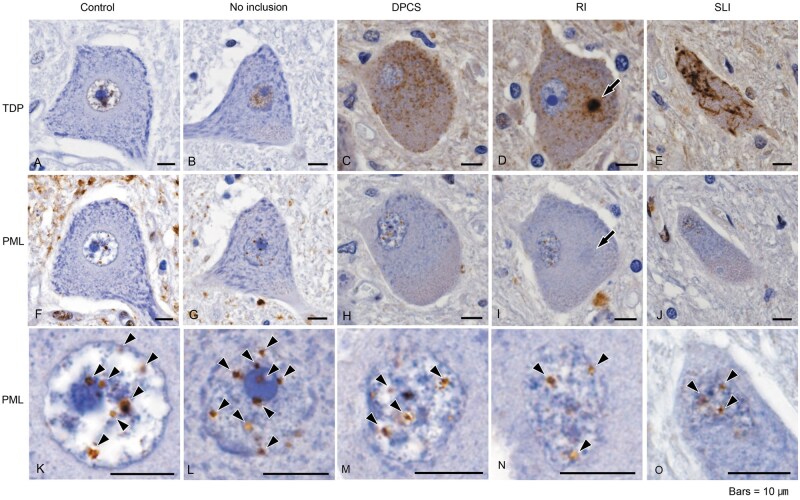
Immunoreactivity of n-TDP-43, p-TDP-43, and PML in serial sections of AHCs in normal controls and patients with ALS. Panels A and F, B and G, C and H, D and I, and E and J represent consecutive sections. Panels K-O provide higher-magnification views of the cell nucleus from F to J. (A, F, K) A normal-appearing AHC with nuclear n-TDP-43-positive granules (A) contains several PML-NBs (F, arrowheads in K). (K) Higher magnification view of the nucleus in F. (B, G, L) An AHC with nuclear n-TDP-43-positive granules (B) contains several PML-NBs (G, arrowheads in L). (L) Higher magnification view of the nucleus in G. (C, H, M) An AHC with diffuse punctate cytoplasmic staining for p-TDP-43 (C) contains a few PML-NBs (H, arrowheads in M). (M) Higher magnification view of the eccentric nucleus in H. (D, I, N) An AHC with a p-TDP-43-positive round inclusion (arrows in D, I) contains few PML-NBs (I, arrowheads in N). (N) Higher magnification view of the eccentric nucleus in I. (E, J, O) An atrophic AHC with p-TDP-43-positive skein-like inclusions (E) contains few PML-NBs (J, arrowheads in O). (O) Higher magnification view of the atrophic and eccentric nucleus in J. Scale bars: 10 μm (A-O). n-TDP, anti-native TAR DNA-binding protein 43; p-TDP: phosphorylated TDP 43, PML: promyelocytic leukemia protein, NB: nuclear bodies, AHC: anterior horn cell.

In the control specimens, most AHCs showed n-TDP-43-positve granular staining (Figure 1A) and several PML-NBs in the nucleus (Figure 1F and K). In the ALS specimens, AHCs without TDP-43-positive inclusions (“no inclusion”) showed n-TDP-43-positve granular staining (Figure 1B), and a few or several PML-NBs in the nucleus (Figure 1G and L). AHCs containing DPCS (Figure 1C), RIs (Figure 1D), and SLIs (Figure 1E) showed depletion of nuclear TDP-43 staining and a few PML-NBs in the nucleus (Figure 1H–J and M–O).

### Quantification of PML-NB numbers

Nuclei were detected in 254 (43.2%) of the 591 AHCs in normal controls (Table 1) versus 379 (42.6%) of the 889 AHCs from patients with ALS. In the nucleated AHCs among the ALS specimens, TDP-43-positive inclusions (no inclusion group) were absent in 189 AHCs; 112 AHCs exhibited DPCS (DPCS group); 60 AHCs had RIs (RI group); and 18 AHCs had SLIs (SLI group).

### Comparison of PML-NB numbers and densities

**Figure 2:**
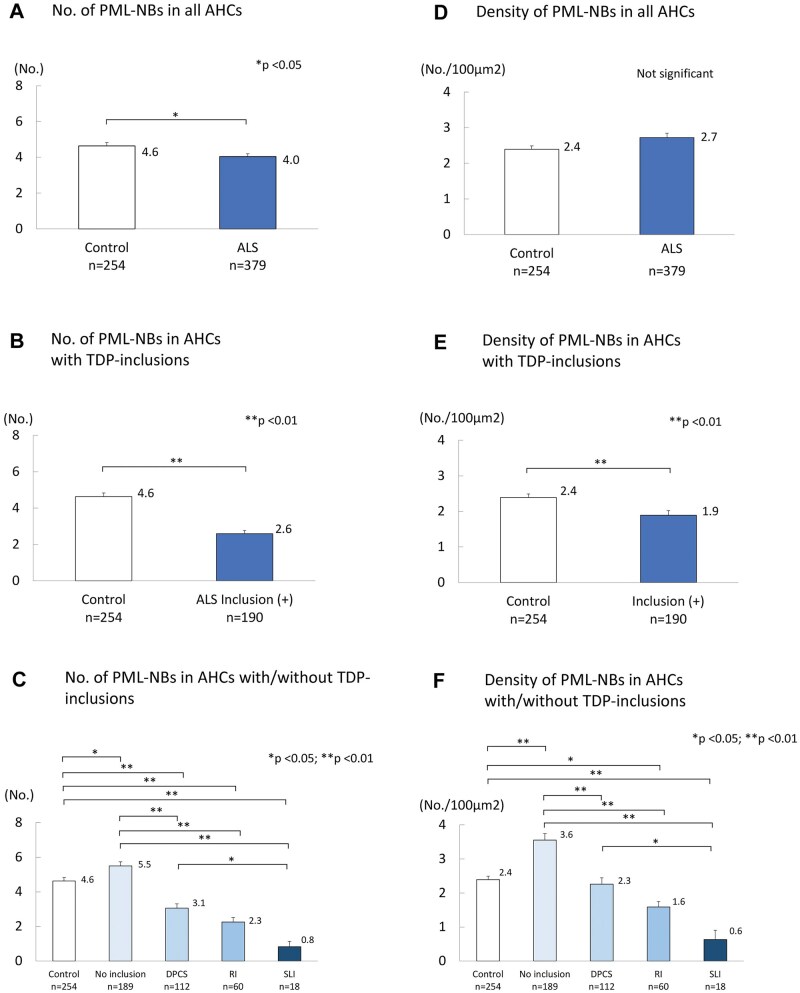
Comparison of the numbers and densities of PML-NBs in AHCs between normal controls and patients with ALS. (A) Comparison of the average numbers of PML-NBs in all AHCs between the control and ALS cohorts. The average number of PML-NBs in all AHCs in the ALS cohort was significantly lower than that in the control cohort (*P* < 0.05). (B) Comparison of the average numbers of PML-NBs in all AHCs in controls and AHCs containing TDP-43-positive inclusions in ALS. The average number of PML-NBs in AHCs containing TDP-43 inclusions in the ALS cohort was significantly lower than that in the control cohort (*P* < 0.01). (C) Comparison of the average numbers of PML-NBs in all AHCs in the controls and AHCs with no inclusions, DPCS, RIs, and SLIs in ALS. The average number of PML-NBs in AHCs without inclusions was significantly higher than that in all AHCs in the controls (*P* < 0.05). The average numbers of PML-NBs in AHCs with DPCS, RIs, and SLIs were significantly lower than those in all AHCs in the controls (*P* < 0.01) and those in AHCs without inclusions in ALS (*P* < 0.01). The average number of PML-NBs in AHCs with SLIs was significantly lower than that in AHCs with DPCS (*P* < 0.05). (D) Comparison of the average densities of PML-NBs in all AHCs in the controls and ALS. An increasing trend was evident in the average density of PML-NBs in all AHCs in ALS, compared with the controls (*P* = 0.051, not statistically significant). (E) Comparison of the average densities of PML-NBs in all AHCs in the controls and AHCs containing TDP-43-positive inclusions in patients with ALS. The average density of PML-NBs in AHCs containing TDP-43 inclusions in ALS was significantly lower than that in the controls (*P* < 0.01). (F) Comparison of the average densities of PML-NBs in all AHCs in the controls and AHCs with no inclusions, AHCs with DPCS, AHCs with RIs, and AHCs with SLIs in the ALS cohort. The average density of PML-NBs in AHCs without inclusions was significantly lower than that in all AHCs in the control cohort (*P* < 0.01). The average densities of PML-NBs in AHCs with RIs (*P* < 0.05) and SLIs (*P* < 0.01) were significantly lower than those in all AHCs in the controls (2.4/100 µm^2^). The average densities of PML-NBs in AHCs with DPCS (2.3/100 µm^2^), RIs (1.6/100 µm^2^), and SLIs (0.6/100 µm^2^) were significantly lower than those in AHCs without inclusions in the ALS cohort (3.6/100 µm^2^) (*P* < 0.01; Fig. 2F). The average density of PML-NBs in AHCs with SLIs was significantly lower than that in AHCs with DPCS (*P* < 0.05). TDP: TAR DNA-binding protein 43, p-TDP: phosphorylated TDP 43, PML: promyelocytic leukemia protein, NB: nuclear bodies, AHC: anterior horn cell, DPCS: diffuse punctate cytoplasmic staining, RIs: round inclusions, SLIs: skein-like inclusions.

The average number of PML-NBs in all AHCs in patients with ALS (4.0) was significantly lower than that in the controls (4.6) (*P* < 0.05; Figure 2A). The average density of PML-NBs in all AHCs in the ALS cohort (2.7/100 µm^2^) appeared to be higher than in the control cohort (2.4/100 µm^2^) (Figure 2D), although the difference lacked significance.

The average number of PML-NBs in AHCs containing TDP-43-positive inclusions (DPCS, RIs, and SLIs) in the ALS group (2.6) was significantly lower than that in the controls (4.6) (Figure 2B, *P* < 0.01). Similarly, the average density of PML-NBs in AHCs containing TDP-43 inclusions in the ALS group (1.9/100 µm^2^) was significantly lower than that in the controls (2.4/100 µm^2^) (*P* < 0.01; Figure 2E).

Interestingly, the average number of PML-NBs in AHCs without inclusions (5.5) was significantly higher than that in all AHCs in the controls (4.6) (*P* < 0.05; Figure 2C). The average numbers of PML-NBs in AHCs with DPCS (3.1), RIs (2.6), and SLIs (0.8) were significantly lower than those in all AHCs in the controls (*P* < 0.01; Figure 2C) and in AHCs without inclusions in ALS (4.6) (*P* < 0.01; Figure 2C). The average number of PML-NBs in AHCs with SLIs (0.8) was significantly lower than that in AHCs with DPCS (3.1) (*P* < 0.05; Figure 2C).

In contrast, the average density of PML-NBs in AHCs without inclusions (3.6/100 µm^2^) was significantly higher than that in all AHCs in the control cohort (2.4/100 µm^2^) (*P* < 0.01; Figure 2F). The average densities of PML-NBs in AHCs with RIs (1.6/100 µm^2^) (*P* < 0.05; Figure 2F) and SLIs (0.6/100 µm^2^) (*P* < 0.01; Figure 2F) were significantly lower than those in all AHCs in the control cohort. The average densities of PML-NBs in AHCs with DPCS (2.3/100 µm^2^), RIs (1.6/100 µm^2^) and SLIs (0.6/100 µm^2^) were significantly lower than those in AHCs without inclusions in ALS (3.6/100 µm^2^) (*P* < 0.01; Figure 2F). The average density of PML-NBs in AHCs with SLIs (0.6/100 µm^2^) was significantly lower than that in AHCs with DPCS (2.3/100µm^2^) (*P* < 0.05; Figure 2F).

### Comparison of somatic, nuclear, and nucleolar areas

The average somatic areas of AHCs without inclusions (1314 µm^2^), AHCs with DPCS (1291 µm^2^), AHCs with RIs (1090 µm^2^) and AHCs with SLIs (986 µm^2^) were significantly smaller than those of AHCs in the control cohort (1874 µm^2^), respectively (*P* < 0.01; Figure S2A). In the ALS cohort, the average somatic area of AHCs with RIs (1090 µm^2^) was significantly smaller than that of AHCs without inclusions (1314 µm^2^) (*P* < 0.05; Figure S2A).

**Figure 3:**
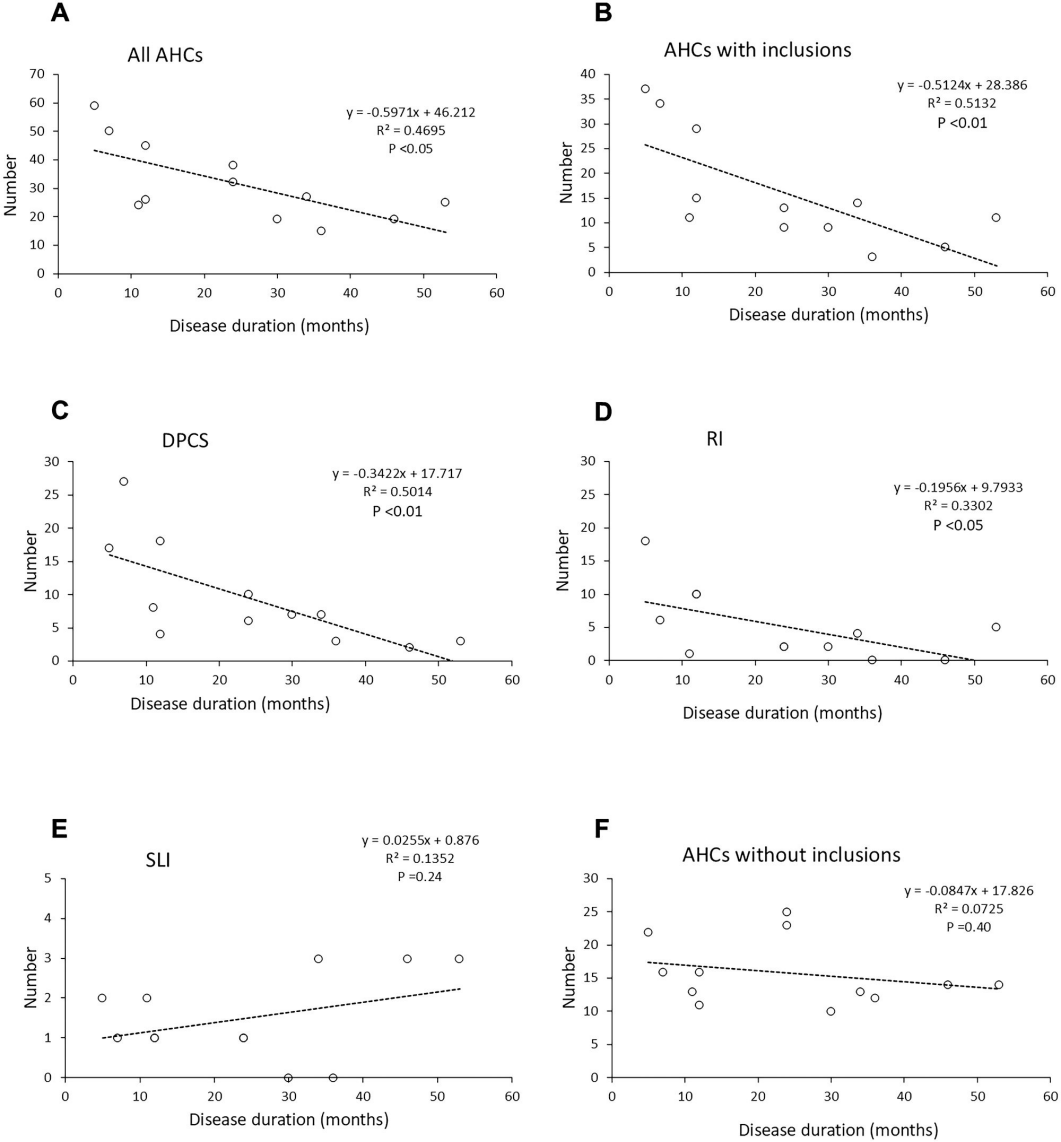
Correlation between disease duration and density of AHCs in ALS. Disease duration was inversely correlated with the number of AHCs (A), AHCs containing any inclusions (B), AHCs with DPCS (C), or AHCs with RIs (D), but not with AHCs with SLIs (E) or AHCs without inclusions (F). ALS: amyotrophic lateral sclerosis, AHC: anterior horn cell, DPCS: diffuse punctate cytoplasmic staining, RIs: round inclusions, SLIs: skein-like inclusions.

The respective average nuclear areas of AHCs without inclusions (175 µm^2^), AHCs with DPCS (150 µm^2^), AHCs with RIs (128 µm^2^) and AHCs with SLIs (108 µm^2^) were significantly smaller than those of controls AHCs (200 µm^2^) (*P* < 0.01; Figure 3B). The average nuclear areas of AHCs with DPCS (150 µm^2^; *P* < 0.05), RIs (128 µm^2^; *P* < 0.01), and SLIs (108 µm^2^; *P* < 0.01) in ALS were significantly smaller than those in AHCs without inclusions (175 µm^2^; Figure S2B).

The average nucleolar areas of AHCs with RIs (16.5 µm^2^) and SLIs (15.0 µm^2^) were significantly smaller than those of control AHCs (24.8 µm^2^) (*P* < 0.01 and *P* < 0.05, respectively; Figure S2C). The average nucleolar areas of AHCs with RIs in ALS were significantly smaller than those of AHCs without inclusions (16.5 µm^2^ vs 21.2 µm^2^; *P* < 0.01).

### Correlation of disease duration with number of AHCs in ALS

Pathologically, sporadic ALS is characterized by the loss of upper and lower motor neurons, with a consistent occurrence of TDP-43 cytoplasmic inclusions. We evaluated the correlation between disease duration and the number of AHCs with or without inclusions. Disease duration was inversely correlated with the number of all AHCs (*P* < 0.05; Figure 3A), AHCs with any inclusion (*P* < 0.01; Figure 3B), AHCs with DPCS (*P* < 0.01; Figure 3C), and AHCs with RIs (*P* < 0.05; Figure 3D), but not with AHCs with SLIs (Figure 3E) or AHCs without inclusions (Figure 3F).

### Immunoelectron microscopy of AHCs using anti-p-TDP-43 antibody

**Figure 4:**
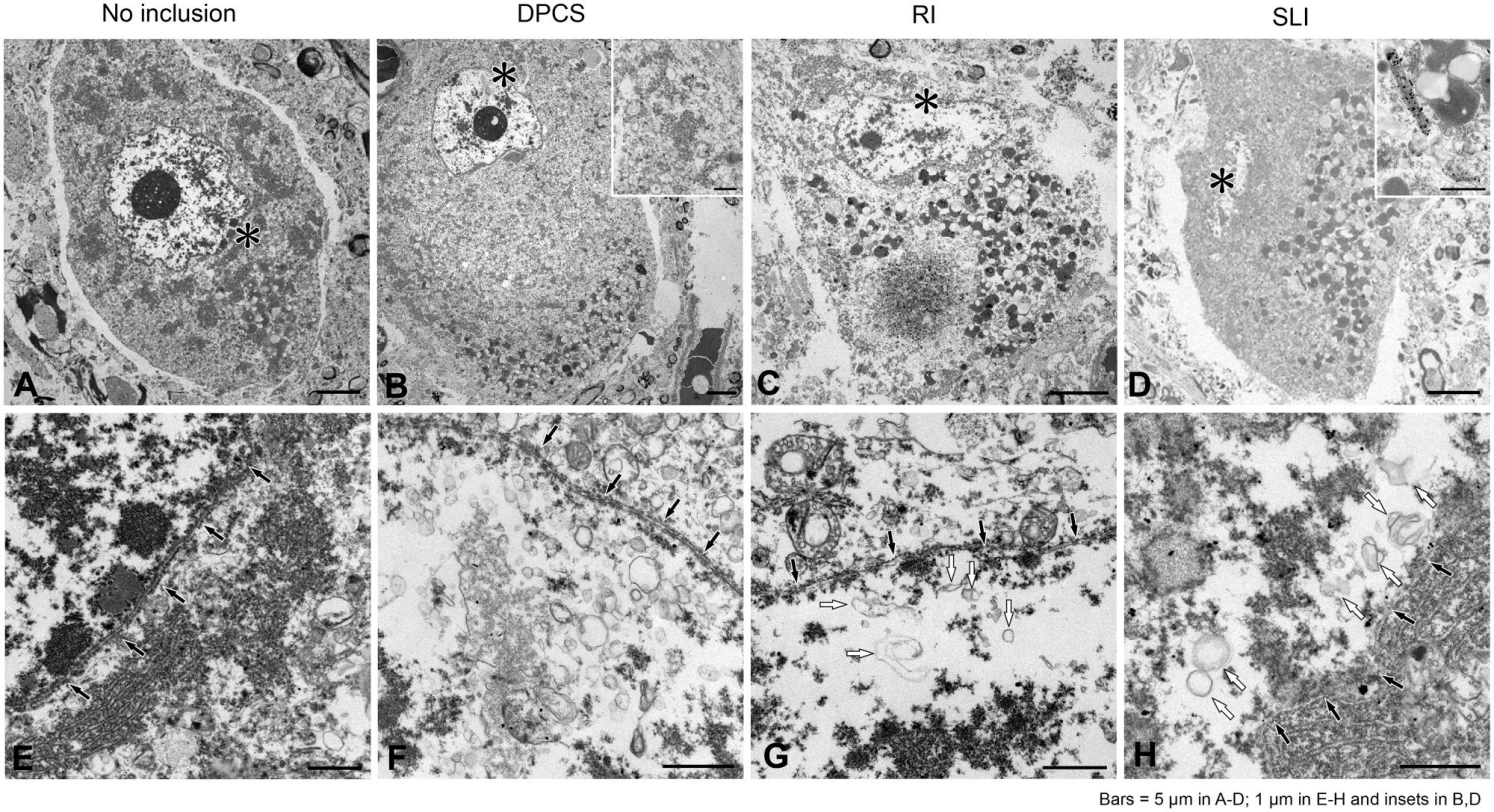
Immunoelectron microscopy of AHCs with or without p-TDP-43 inclusions in ALS using anti-p-TDP-43 antibody. (A, E) An AHC without inclusions (A). (E) A higher-magnification view of the area indicated by the asterisk in (A) showing no vacuoles in the nucleoplasm enveloped by nuclear membrane (black arrows). (B, F) An AHC with p-TDP-43-positive DPCS (B, insert). (F) A higher-magnification view of the area indicated by the asterisk in (B) showing many vacuoles of various sizes in the nucleoplasm enveloped by nuclear membrane (black arrows). (C, G) An AHC with p-TDP-43-positive RIs (C). (G) A higher-magnification view of the area indicated by the asterisk in (C) showing some vacuoles of various shapes (white arrows) in the nucleoplasm enveloped by nuclear membrane (black arrows). (D, H) An AHC with p-TDP-43-positive SLIs (D, insert). (H) A higher-magnification view of the area indicated by the asterisk in (D) showing some vacuoles of various shapes in the nucleoplasm (white arrows) enveloped by nuclear membrane (black arrows). Scale bars: 5 μm (A-D); 1 μm (E-H). p-TDP: phosphorylated TAR DNA-binding protein 43, ALS: amyotrophic lateral sclerosis, AHC: anterior horn cell, DPCS: diffuse punctate cytoplasmic staining, RIs: round inclusions, SLIs: skein-like inclusions.

To analyze alterations in the nuclei and PML-NBs in AHCs, we scrutinized AHCs with or without p-TDP-43 inclusions. Vacuoles were absent in the nucleoplasm of AHCs without inclusions (Figure 4A and E). In contrast, vacuoles of varying sizes and shapes were observed in the nucleoplasm AHCs with p-TDP-43 inclusions (Figure 4B–D and F–H). To evaluate the relationship between intranuclear vacuoles and p-TDP-43 inclusions, we ultrastructurally examined 26 AHCs with and without inclusions (Table 2). Intranuclear vacuoles were present in 14 AHCs containing p-TDP-43-positive inclusions but were absent in 1 AHC containing p-TDP-43-positive inclusions. In contrast, 11 AHCs without inclusions did not exhibit intranuclear vacuoles. The formation of p-TDP-43-positive inclusions bore a significant relationship with the appearance of intranuclear vacuoles (*P* < 0.01, Fisher exact test).

**Table 2.** Relationship between intranuclear vacuoles and p-TDP-43-positive inclusions in anterior horn cells.

	p-TDP-43-positive inclusions (+)	No inclusions	Total
Intranuclear vacuoles (+)	14	0	14
Intranuclear vacuoles (-)	1	11	12
Total	15	11	26

## DISCUSSION

### Changes in the numbers of PML-NBs

In the present study, we demonstrated that the number of PML-NBs in all AHCs of patients with sporadic ALS was significantly lower than that in the AHCs of normal controls. This finding is similar to that reported in familial ALS by Antoniani et al.^15^ We further showed that the number of PML-NBs in AHCs containing TDP-43 inclusions was significantly lower than that in the controls. Moreover, the number of PML-NBs was significantly lower in AHCs with DPCS, RIs, and SLIs compared with all control AHCs. The density of PML-NBs in AHCs with DPCS, RIs, and SLIs also approximated the trend in the number of PML-NBs, confirming the reliability of this phenomenon. Antoniani et al reported that motor neurons harboring either p-TDP-43 or fused in sarcoma (FUS) aggregates specifically showed diminished numbers of PML-NBs compared with adjacent motor neurons devoid of any aggregates in familial ALS.^15^ Similarly, we found that the number of PML-NBs was also lower in AHCs with TDP-43 aggregates in sporadic ALS, indicating that the number of PML-NBs may gradually decline following maturation of TDP-43 inclusions (Figure 2C). In contrast, the number of PML-NBs in normal-looking AHCs without inclusions in patients with ALS was significantly higher than that in control AHCs. This may reflect a compensatory response aimed at preserving cellular physiological function against the milieu of precursor and early pathological conditions in ALS neurons. As observed in normal controls, PML-NBs exist in cells grown under physiological conditions. However, upon exposure to various stresses, the number of PML-NBs increases substantially,^20^ supporting the finding that the number of PML-NBs is elevated in normal-appearing AHCs without TDP-43 inclusions in ALS.

### TDP-43 nuclear depletion associated with changes in the numbers of PML-NBs

Although the cause underlying the decline in PML-NB numbers in ALS is currently unclear, Antoniani et al indicated the existence of crosstalk between the cytoplasmic and nuclear protein quality control systems; the alterations in these may contribute to SG accumulation and cytoplasmic protein aggregation in ALS.^15^ PML-NBs have been implicated in cargo protein modification or degradation, as well as sequestration within the nucleus,^13^ and may facilitate the clearance of TDP-43 and FUS.^21^ Moreover, in mouse and cell culture models of spinocerebellar ataxia type 1 (SCA1), disruption of PML-NBs led to increased accumulation of nuclear ataxin-1, a pathogenic protein in SCA1, owing to impaired removal of misfolded proteins.^22^ These findings suggest that loss of physiological PML-NB function contributes to protein aggregation. Based on this concept, we interpret our finding of a gradual decrease in the number and density of PML-NBs following the maturation of TDP-43 inclusions as reflecting disease progression in sporadic ALS due to the exhaustion of the proteostatic capacity of PML-NBs.

### Intranuclear membranous vacuoles in the AHCs with TDP-43 inclusions

Immunoelectron microscopy demonstrated that intranuclear membranous vacuoles were present consistently in AHCs with p-TDP-43 inclusions in ALS but not in ALS AHCs without inclusions. Membranous profiles acceptable as sections through the lamellae, tubules, and vesicles have at times been convincingly demonstrated, as has continuity between the inner nuclear membrane, in various disease conditions.^23^ These profiles are thought to represent nuclear lipid droplets. Nuclear lipid droplets have been observed sporadically inside the nucleus of some mammalian cells^24^; they originate from the nuclear envelope.^25^ PML is reportedly involved in the formation of nuclear lipid droplets.^24^ Recently, ALS has been linked to the formation of lipid droplets.^26^ Some studies have suggested that TDP-43 dysfunction participates directly in the alteration of lipid regulation and obesity, implying the general role of TDP-43 in lipid metabolism.^27^ Considering the presence of nuclear atrophy in AHCs in patients with ALS in our study, the presence of intranuclear membranous profiles might be one of the pathological changes in AHCs in ALS, potentially indicative of disrupted lipid metabolism.

### Pathological changes in AHCs containing TDP-43 inclusions may be irreversible

AHCs with TDP-43-positive inclusions showed fewer or no PML-NBs, nuclear depletion of TDP-43 and obvious somatic and nuclear atrophy with alteration of intracellular organelles (eg, Nissl bodies, Golgi apparatus^28^) and intranuclear membranous profiles. In addition, nucleolar atrophy was evident in the AHCs with TDP-43-positive RIs and SLIs. Moreover, significant negative correlations were observed between disease duration and the number of AHCs with TDP-43 inclusions. These findings suggest that AHCs with TDP-43-positive cytoplasmic inclusions are dying neurons. In contrast, no significant correlations were observed between disease duration and the number of AHCs without TDP-43 inclusions, suggesting that AHCs without inclusions may survive.

This study has some limitations. First, we did not perform immunohistochemical staining for ubiquitin or SUMO. In this study, staining for nTDP-43, PML, and pTDP-43 was conducted using 3 consecutive sections of human spinal cord. Because a portion of the same anterior horn cell nucleus (maximum diameter approximately 10 µm) was present across these three 4-µm-thick sections, we were able to examine the relationship between the number of PML nuclear bodies and TDP-43–positive inclusions. Unfortunately, further expansion of this approach is limited by methodological constraints, including the small nuclear diameter and the technical difficulty of preparing a larger number of consecutive sections. We also attempted multiplex fluorescence immunostaining; however, this method failed to detect TDP-43 puncta in DPCS due to strong lipofuscin autofluorescence. Assessing changes in the ubiquitylation or SUMOylation of PML-NBs would provide valuable information for future study. Although we observed concurrent loss of PML-NBs and the emergence of nuclear vacuoles in cells with TDP-43 pathology, direct ultrastructural colocalization of PML and TDP-43 with nuclear lipid droplets was not performed. Single- or double-label immunoelectron microscopy would be required to establish direct spatial relationships.

In conclusion, PML-NBs are involved in the disease process of not only familial ALS but also sporadic ALS.

## Supplementary Material

nlag029_Supplementary_Data
